# Bacterial Density and Biofilm Structure Determined by Optical Coherence Tomography

**DOI:** 10.1038/s41598-019-46196-7

**Published:** 2019-07-05

**Authors:** Jiapeng Hou, Can Wang, René T. Rozenbaum, Niar Gusnaniar, Ed D. de Jong, Willem Woudstra, Gésinda I. Geertsema-Doornbusch, Jelly Atema-Smit, Jelmer Sjollema, Yijin Ren, Henk J. Busscher, Henny C. van der Mei

**Affiliations:** 1 University of Groningen and University Medical Center Groningen, Department of Biomedical Engineering, P.O. Box 196, 9700 AD Groningen, The Netherlands; 2 University of Groningen and University Medical Center Groningen, Department of Orthodontics, Hanzeplein 1, 9713 GZ Groningen, The Netherlands

**Keywords:** Applied optics, Biophysics, Biofilms

## Abstract

Optical-coherence-tomography (OCT) is a non-destructive tool for biofilm imaging, not requiring staining, and used to measure biofilm thickness and putative comparison of biofilm structure based on signal intensity distributions in OCT-images. Quantitative comparison of biofilm signal intensities in OCT-images, is difficult due to the auto-scaling applied in OCT-instruments to ensure optimal quality of individual images. Here, we developed a method to eliminate the influence of auto-scaling in order to allow quantitative comparison of biofilm densities in different images. Auto- and re-scaled signal intensities could be qualitatively interpreted in line with biofilm characteristics for single and multi-species biofilms of different strains and species (cocci and rod-shaped organisms), demonstrating qualitative validity of auto- and re-scaling analyses. However, specific features of pseudomonas and oral multi-species biofilms were more prominently expressed after re-scaling. Quantitative validation was obtained by relating average auto- and re-scaled signal intensities across biofilm images with volumetric-bacterial-densities in biofilms, independently obtained using enumeration of bacterial numbers per unit biofilm volume. The signal intensities in auto-scaled biofilm images did not significantly relate with volumetric-bacterial-densities, whereas re-scaled intensities in images of biofilms of widely different strains and species increased linearly with independently determined volumetric-bacterial-densities in the biofilms. Herewith, the proposed re-scaling of signal intensity distributions in OCT-images significantly enhances the possibilities of biofilm imaging using OCT.

## Introduction

Biofilms mostly grow on surfaces in aqueous environments, that range from marine and industrial to biomedical environments^1,2^. Biofilms have complicated, heterogeneous structures that influence their resistance to mechanical or chemical challenges^3–5^, such as fluid shear or detergents and other antimicrobials. However, microscopic methods available for non-destructive analysis of biofilm structure, while not requiring staining, are scarce and frequently only provide low resolution images covering a small field of view. By consequence, these methods yield simple morphological characteristics, such as substratum surface coverage or thickness^6,7^. Low load compression testing and analysis of stress relaxation over time has also been suggested as a means to evaluate biofilm structure over a large surface area^8^, but does not provide detailed structural information. Magnetic resonance imaging (MRI) or magnetic resonance microscopy can track free water molecules in biofilm structures on non-metal surfaces with a limited resolution^9^, as opposed to bound, interfacial water present on any surface^10^.

Optical coherence tomography (OCT) is a rapid, real-time, *in situ* and non-destructive imaging method, not requiring any staining, and is widely used in biofilm research to measure biofilm thickness and morphology^11–16^. OCT is based on light scattering by a substratum surface, including biofilms grown on these surfaces. Accordingly, objects with higher light scattering will yield higher signal intensities, than objects with lower scattering, yielding a signal intensity distribution in OCT images of a biofilm, usually visualized as different shades of white-black in OCT images. In order to measure biofilm thickness from an OCT image, the whiteness of the biofilm reflecting high signal intensities has to be distinguished from the relatively black background of its aqueous environment, which can be done by proper thresholding^17^ to define the exact position of the biofilm surface. However, since the biofilms itself also contains water, relatively black pixels reflecting low signal intensities are left inside the biofilm which have been tentatively ascribed to water-filled pores^18^. In addition, limited attention has been given to relatively white pixels in biofilm images with a signal intensity above the threshold and how they possibly reflect different biomass components, such as bacteria and aggregates of water-insoluble extracellular polymeric substances (EPS)^19^. Haisch & Niessner^20^ were the first to introduce a whiteness scale bar in OCT biofilm imaging, representing the signal intensity of the scattered light and suggested signal intensities to increase with volumetric bacterial density in a biofilm, but without supporting data. Signal intensity increases in OCT images of biofilms have been observed after antimicrobial treatment of a biofilm, without rigorous interpretation^21^. Signal intensities and comparisons of OCT images may be influenced by the auto-scaling applied in OCT instruments to ensure that each image contains an intensity distribution that covers the entire signal intensity range available. Auto-scaling of signal intensities in biofilm images has been applied to staphylococcal biofilms on stainless steel surfaces, but only yielded a qualitative relation between volumetric bacterial density in a biofilm and biofilm intensity in OCT images^22^, presumably due to differences in intensity distribution across different individual images created by the auto-scaling.

The objective of this study was to develop a method to analyze and quantitatively compare the intensity distribution in different OCT images of biofilms from which biofilm structure and volumetric bacterial density can be derived. A re-scaling method was developed that effectively removes the influence of auto-scaling across different images and allows to quantitatively relate volumetric bacterial density with biofilm signal intensity in OCT images. Auto- and re-scaling methods, will be applied on single- and multi-species (or sometimes called “mixed-species” or “co-culture”) biofilms composed of Gram-positive and Gram-negative strains (cocci and rod-shaped organisms) with known properties, such as EPS production and ability to (co-)aggregate, as grown on different substratum surfaces under widely different conditions (static, under flow or in a constant depth film fermenter). Correspondence between expectations based on known properties of the biofilm forming strains with conclusions drawn from the auto- or re-scaling methods and enumeration of the number of bacteria in a biofilm, will be taken as a validation for the respective methods (see Table 1 for an overview of the biofilm cases involved).

**Table 1:** Summary of the different biofilm cases involved in this study, specifying the strains and substratum materials, growth conditions and media as well as the characteristics of the biofilms, expected on the basis of literature.

Case	Strain	Material	Growth condition	Growth medium	Expected biofilm characteristics
Case 1	*S. epidermidis* 252	Stainless steel	Static	TSB	Higher EPS production by *S. epidermidis* ATCC 35984 than *S. epidermidis* 252^22,38^
	*S. epidermidis* ATCC 35984				
Case 2	*S. mutans* UA159	Polystyrene	Static	BHI + 0.5% sucrose	More water-filled channels upon higher sucrose levels^31^
				BHI + 1.0% sucrose	
Case 3	*P. aeruginosa* ATCC 39324	Stainless steel	Constant depth film fermenter	LB	Higher EPS production for biofilms grown in ASM than in LB^28^
				ASM	
Case 4	*S. oralis* J22	Glass	Flow	BHI^+^	More compact multi-species biofilms due to co-aggregation^32^ as compared with mono-species biofilms
	*A. naeslundii* T14V-J1			BHI^+^	
	Multi-species *S. oralis* J22 and *A. naeslundii* T14V-J1			BHI^+^	

## Materials and Methods

### Bacterial strains and growth conditions

Non-EPS producing *Staphylococcus epidermidis* 252 and EPS producing *Staphylococcus epidermidis* ATCC 35984 were isolated from the stool of a patient and a catheter-associated sepsis of a patient, respectively^23^. *Streptococcus mutans* UA159 (ATCC 700610) was isolated from a patient with active dental caries^24^, while also *Streptococcus oralis* J22 and *Actinomyces naeslundii* T14V-J1 (rod-shaped) were both isolated from the human oral cavity^25^. *Pseudomonas aeruginosa* ATCC 39324 (rod-shaped) was isolated from sputum from a cystic fibrosis patient^26^. Each strain was inoculated from a single colony taken from a blood agar plate, in a 10 mL pre-culture and grown for 24 h at 37 °C. This pre-culture was inoculated in a 200 mL main culture, grown for 16 h at 37 °C. The culture medium was Tryptone Soya Broth (TSB, Oxoid, Basingstoke, UK) supplemented with 0.25% D(+)glucose (C_6_ H_12_O_6_, Merck, Darmstadt, Germany) and 0.5% NaCl (Merck) for *S. epidermidis* 252 and *S. epidermidis* ATCC 35984; Brain Heart Infusion (BHI, Oxoid, Basingstoke, UK) supplemented with 0.5% or 1% (w/v) sucrose for *S. mutans* UA159; TSB for *P. aeruginosa* ATCC 39324; Todd Hewitt broth (THB, Oxoid) for *S. oralis* J22; BHI^+^ (BHI supplemented with 1 g L^−1^ yeast extract, 50 mg L^−1^ hemin and 1 mg L^−1^ menadion) for *A. naeslundii* T14V-J1. *A. naeslundii* T14V-J1 was cultured in an anaerobic cabinet, *S. mutans* UA159 in 5% CO_2_ while all other strains were cultured in ambient air. Bacteria were harvested from their main cultures by centrifugation at 5000 g, 10 °C, and washed twice with buffer, after which bacteria were enumerated using a Bürker-Türk counting chamber.

### Biofilm formation

Biofilms of the different strains were grown on different substrata according to previously used protocols that will be briefly repeated for clarity for the different cases (see also Table 1). *S. epidermidis* ATCC 35984 and *S. epidermidis* 252 biofilms were grown on sterile, stainless steel 304 (SS) surfaces (15 × 15 × 1 mm) coated with 10% fetal bovine serum (FBS) under static conditions^22^. After allowing bacterial adhesion for 1 h from a 1 × 10^9^ mL^−1^ bacterial suspension in TSB, the suspension was replaced by medium without staphylococci and biofilms were grown for 48 h at 37 °C, after which biofilms were washed with reduced transport fluid^27^ (Rundell *et al*., 1973) for subsequent experiments.

For *S. mutans* UA159 biofilms, a buffer suspension (1 mM CaCl_2_, 2 mM potassium phosphate, 50 mM KCl, pH 6.8, bacterial concentration of 3 × 10^8^ mL^−1^) was added in a 24 wells polystyrene plate (Greiner Bio-One GmbH, Frickenhausen, Germany) under static conditions for 2 h at 37 °C under 5% CO_2_ to allow streptococci to adhere. After adhesion, the buffer was removed and carefully washed with buffer, 1 mL BHI with 0.5% or 1% sucrose (w/v) was added and the plate was incubated for 24 h under static conditions after which biofilms were washed with buffer for subsequent OCT experiments.

Biofilms of *P. aeruginosa* ATCC 39324 were grown on SS disks in a constant depth film fermenter (CDFF)^28^ at 37 °C. Sample holders were recessed to a well-depth of 100 µm and placed into so-called pans (5 sample holders per pan), of which 15 pans could be placed in the CDFF turntable. Two hundred mL bacterial (5 × 10^7^ mL^−1^) suspension in TSB was drop-wise added on the turntable during 1 h, while the turntable was rotating at 3 revolutions per minute and bacterial suspension was distributed over the sample holders in the various pans by a Teflon scraper-blade. After 1 h, rotation was stopped for 30 min to allow bacteria to adhere to the stainless steel surfaces. Next, rotation was continued and Luria Bertani (LB, Sigma-Aldrich, St Louis, MO, USA) broth or artificial sputum medium^29^ (ASM, per liter: 4 g DNA, 5 g mucin, 5 mL egg yolk emulsion, 5 g NaCl, 2.2 g KCl, 5 g amino acids, pH 7.0) was drop-wise added at a flow rate of 15 mL h^−1^ for 18 h, while the scraper blade removed biofilm growing above the wells in order to grow constant depth biofilms with 100 μm thickness. After 18 h, the stainless disks with biofilms were washed and submerged in phosphate buffered saline (PBS, 10 mM potassium phosphate, 150 mM NaCl, pH 7.0) for subsequent experiments.

Multi-species biofilms of the coccal *S. oralis* J22 and rod-shaped *A. naeslundii* T14V-J1 co-adhering pair and each single strain were grown on a salivary conditioning film covered glass surface, constituting the bottom plate of a parallel plate flow chamber (17.5 × 1.7 × 0.075 cm) at 37 °C^30^. *A. naeslundii* T14V-J1 was suspended in buffer (1 mM CaCl_2_, 2 mM potassium phosphate, 50 mM KCl, pH 6.8) supplemented with 2% BHI^+^ to a concentration of 1 × 10^8^ mL^−1^, while *S. oralis* J22 was suspended to a concentration of 3 × 10^8^ mL^−1^. Briefly, for single strain biofilms, bacterial suspension was perfused through the flow chamber at 10 s^−1^ for 2 h after which the buffer was replaced by growth medium for overnight growth at 3 s^−1^. For biofilms of the co-adhering pair, *A. naeslundii* T14V-J1 was adhered first for 15 min at 10 s^−1^, followed by rinsing and co-adhesion of *S. oralis* J22 for 2 h and buffer was replaced by 50% diluted BHI^+^ in buffer for 16 h growth at 3 s^−1^. After growth, the flow chamber was rinsed with buffer and biofilms were imaged using the OCT while in the flow chamber and subsequently removed for further experiments.

### OCT measurements and signal intensity analysis of OCT 2D images

Biofilms were imaged using an OCT Ganymede II (Thorlabs Ganymede, Newton, NJ, USA) with a 930 nm center wavelength white light beam and a Thorlabs LSM03 objective scan lens, able to provide a maximum scan area of 100 mm^2^. Imaging frequency was 30 kHz with a sensitivity of 101 dB and the refractive index of biofilm was set as 1.33, equal to the one of water. 2D images had fixed 5000 pixels with variable pixel size, depending on magnification in the horizontal direction, while containing a variable number of pixels with 2.68 µm pixel size in the vertical direction. Images were created by the OCT software (ThorImage OCT 4.1) using 32 bit data, but methods outlined are equally applicable to 8 or 16 bit images. The back-scattered light from a sample was captured by a CCD camera and the analogue voltage output of the camera was set such that the average light signal intensity over an entire 2D image was zero. Next, signal intensity was expressed in decibel units with respect to an internal reference signal intensity, generated in the OCT. Decibel units digitized over a discrete intensity scale ranging from 0 a.u. (arbitrary units, with 0 a.u. reflecting the darkest pixel, i.e. the lowest signal intensity in the image) to 255 a.u. (whitest pixel. i.e. the highest signal intensity in the image) in order to generate a 2D “auto-scaled” image. Thus obtained signal intensities associated with each pixel were collected from ten randomly chosen 2D images of each biofilm (Labview 2014, National Instrument, Austin, Texas, USA) and computer-stored for further analysis. The thickness of the biofilms was calculated after Otsu^17^ thresholding of a biofilm image, while averaging biofilm thicknesses obtained over ten images.

In addition to imaging biofilms, bacterial suspensions of the different strains in an aqueous medium over a wide range of bacterial concentrations in a 24 wells plate without a cover, were also imaged using OCT. Bacterial concentrations were quantitated by their optical density OD_600nm_ in polystyrene cuvettes after appropriate dilution to different concentrations. PBS was used as a control in OCT (no bacteria) and its optical density subtracted from all densities measured on a bacterial suspension. The bacterial concentration in aqueous medium was subsequently related with signal intensities in OCT images.

### Calculation of volumetric bacterial density

For the determination of the volumetric bacterial density in the different biofilm cases, biofilms were removed from the different sample surfaces with a sterile disposable cell scraper (Merck, Darmstadt, Germany), after which bacteria were dispersed in buffer by vortexing. The dispersed biofilms were sonicated 3 times on ice at 30W (Vibra Cell Model 375, Sonics and Materials Inc., Danbury, CT, USA) for 10 s each time with 30 s interval to obtain single bacteria. Then, the bacterial concentrations in the suspensions were enumerated in a Bürker–Türk counting chamber and the volumetric bacterial density in the biofilms was calculated by dividing the total number of bacteria on a sample by the total biofilm volume, i.e. the biofilm covered substratum surface area multiplied by the biofilm thickness, as determined using OCT.

## Results and Discussion

### OCT imaging

**Figure 1:**
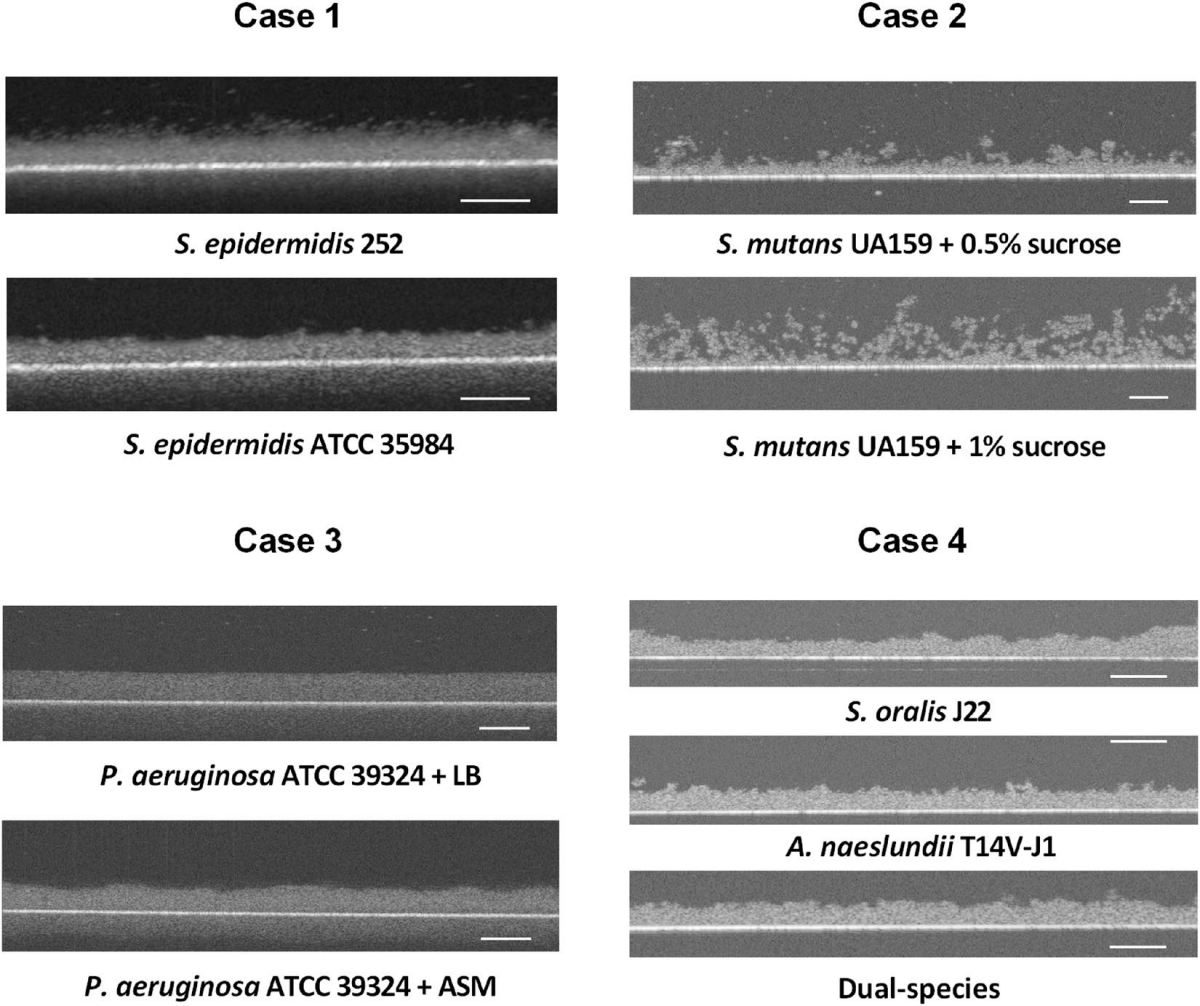
2D cross-sectional OCT images of the four case biofilms evaluated. The scale bars indicate 100 µm.

First, 2D OCT images were made of the biofilms (Fig. 1), representing the four different cases summarized in Table 1. All four cases showed distinctly different features within 2D cross-sectional OCT images of the different biofilms. For statically grown staphylococcal biofilms, EPS producing *S. epidermidis* ATCC 35984 possessed a smoother biofilm surface than its non-EPS producing counterpart. Statically grown *S. mutans* biofilm surfaces appeared quite rough and had clearly enhanced porosity when grown at the higher sucrose concentration. Both CDFF grown *P. aeruginosa* biofilms had the same thickness and were relatively smooth at their surfaces, due to the action of the CDFF scraper.

The *S. oralis* single-species biofilm grown under flow, appeared smoother than the *S. mutans* biofilms grown statically, while single-species biofilms of rod-shaped *A. naeslundii* and *A. naeslundii* containing multi-species biofilms looked rougher despite also being grown under flow.

**Figure 2:**
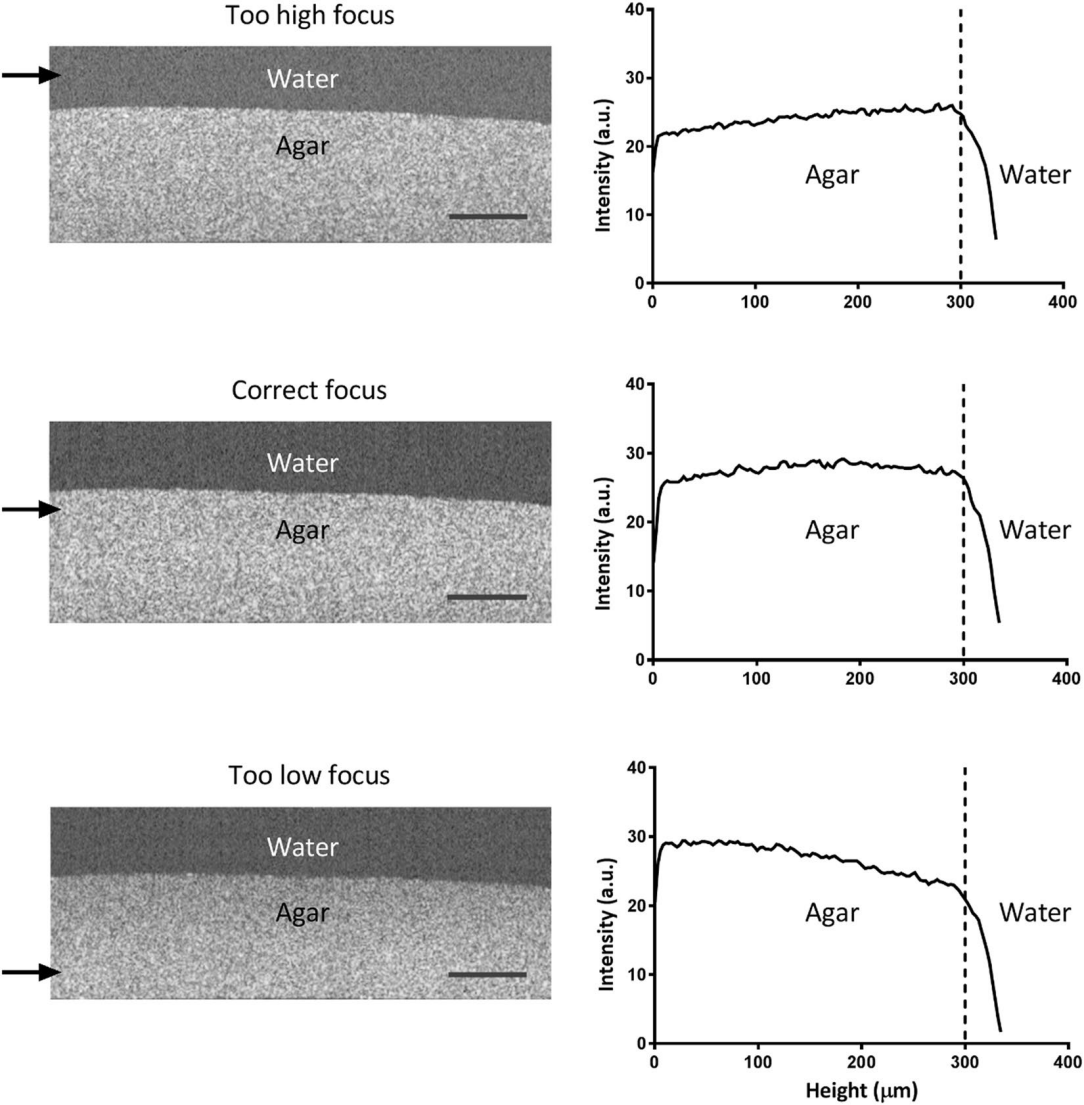
Influence of the OCT focus point on the signal intensity distribution across a 2D cross-sectional image of a homogeneous agar layer. OCT images represent the cross-sectional view of an MRS agar layer at different focus points, indicated by the arrows. For each image, the average signal intensity is presented as a function of the height in the agar layer. Scale bars equal 200 µm. a.u. denotes “arbitrary unit”.

Most noticeably, whereas the aqueous environments above the different case biofilms appeared relatively black in OCT images (auto-scaled signal intensity of the blackest pixel 0 a.u.), the substratum surfaces, i.e. either stainless steel, polystyrene or glass, appeared as the most intense signal region (auto-scaled signal intensity of the whitest pixel 255 a.u.). Despite the auto-scaling, the image examples in Fig. 1 represent different signal intensities for water above the biofilms, with the water above the multi-species biofilms composed of cocci and rod-shaped organisms, yielding the highest signal intensity (case 4). Although there evidently is water above all biofilms, the region above the biofilms appears to yield the lowest signal intensity above the examples of staphylococcal biofilms (case 1). This represents the main problem in comparing OCT images of different biofilms or on different substrata based on signal intensities and associated whiteness scale bars. The first reason that water yields a different signal intensity above different biofilms is due to the occurrence of single, extremely black pixels, invisible by the naked eye in an image due to their small size, but determinant for the auto-scaling. A second reason is that proper focusing is extremely important. According to the Thorlab OCT manual, the focus point should be set just below the surface of the object to be imaged, which is not trivial for a biofilm surface. This is illustrated in Fig. 2, showing the effect of slightly miss-focusing too high above, or too low inside a 300 µm thick homogeneous MRS agar layer. A constant signal intensity across the entire thickness of the agar with a relatively black background (signal intensity around 28 a.u.) is obtained for the “correct” focus point just below the agar surface. Purposely focusing too high or too low, not only led to signal intensities that varied over the thickness of the homogeneous agar layer, but also to a different signal intensity of the water above the agar, similar to the differences in signal intensities seen above the different biofilm cases in Fig. 1. The constant intensity over the depth of a 300 µm thick homogeneous MRS agar layer, also implies that signal intensity analysis along the depth of a biofilm is not influenced by the decline of the signal-to-noise ratio (also called “drop-off”)^16^ along the optical axis of the OCT device over the thickness of the biofilms studied.

### Re-scaling of the signal intensity distribution

**Figure 3:**
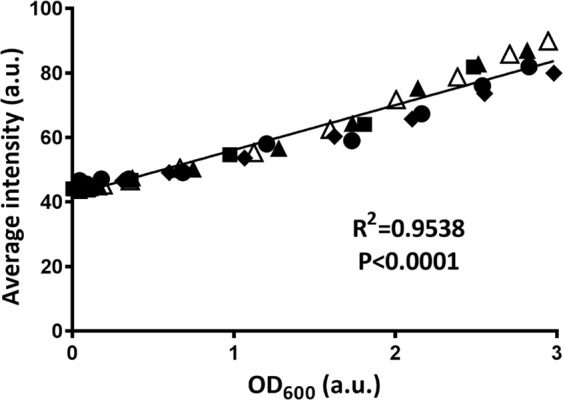
Average intensity (auto-scaled) in OCT images of bacterial suspensions in PBS as a function of the optical density, OD_600,_ of the suspensions. Data involve different concentrations of widely different strains and species (*S. epidermidis* 252, *S. epidermidis* ATCC 35984, *P. aeruginosa* 39324, *S. mutans* UA159 or *S. oralis* J22). Different strains are indicated by different symbols, but not further specified due to overlap of data points at similar optical densities. Drawn lines represent the best fit to an assumed linear relation with correlation coefficient R^2^ and significance of the slope P indicated.

**Figure 4:**
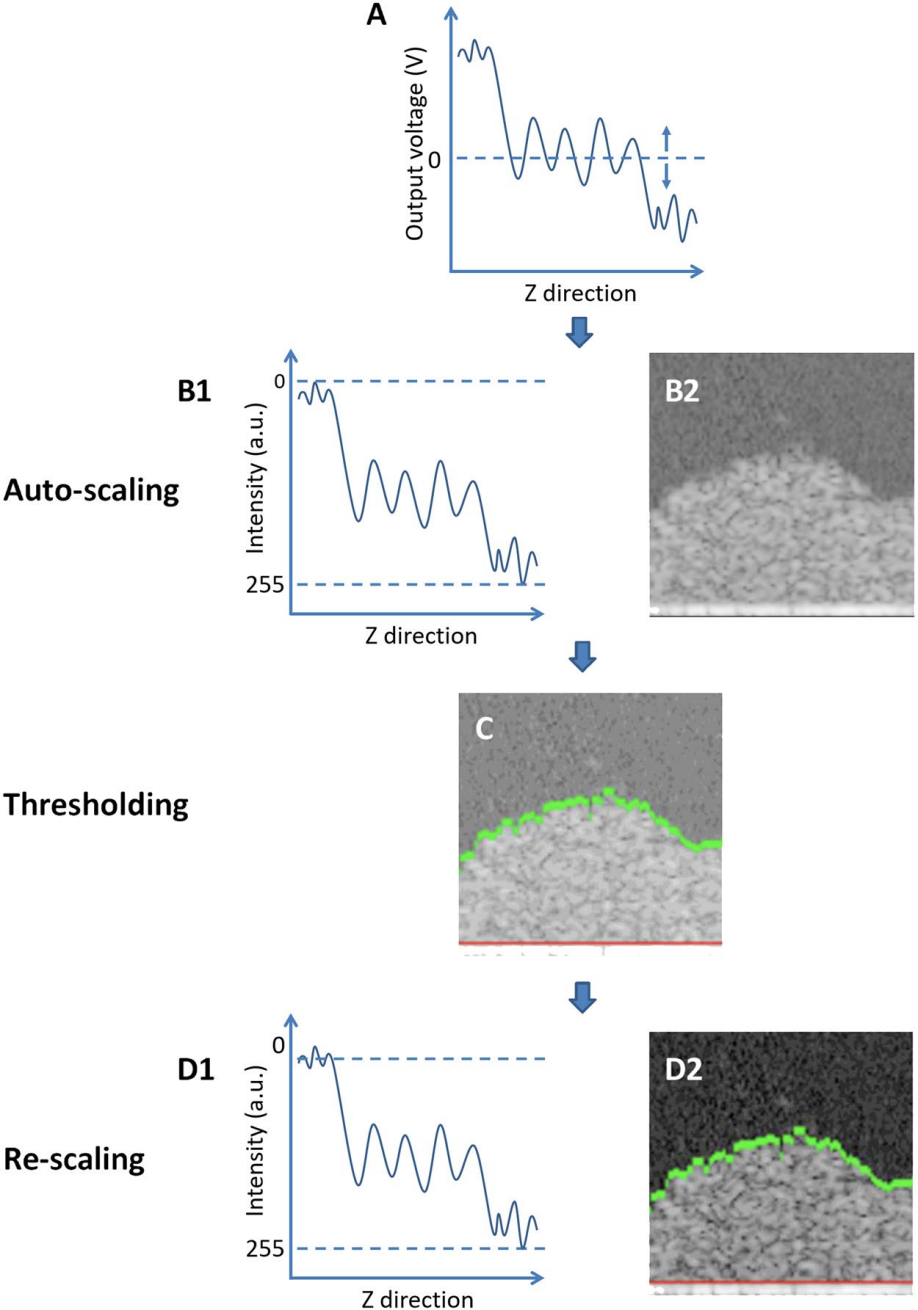
Auto- and re-scaling based analyses of 2D cross-sectional OCT images. (**A**) The back-scattered light from the measured sample is collected by the OCT camera and its intensity outputted as analogue voltage. Panel A showed an example of the output voltage in the Z-direction perpendicular to the substrate. Note the OCT adjust the level of zero voltage to ensure zero average output over an entire image. **(B**) In the subsequent auto-scaling process, voltage is firstly expressed in decibel units with respect to a reference intensity provided by the instruments after which the decibel scale is digitized in 256 discrete values from to 0 and 255 a.u. (panel B1) to yield the image provided in panel B2. Intensity distribution in auto-scaled images is created by the OCT instrument to ensure optimal quality of an individual image. (**C**) Next, Otsu thresholding^17^ is applied on the OCT image to determine the biofilm surface (green line), while the substratum surface is visually identified based on the abrupt increase in intensity as compared with the biofilm interior. In order to avoid a potential impact of the intensity of the substratum material on the intensity of the biofilm, for calculational purposes the substratum surface was positioned 3 µm above the visually identified surface (red line). (**D**) In the proposed re-scaling process of OCT images, the average auto-scaled signal intensity above the biofilm surface, as identified by Otsu thresholding, is given a new intensity value of 0 a.u., while the separately measured, average intensity of a biofilm-free substratum is used and adjusted to an intensity value of 255 a.u. (panel D1). A new OCT image is subsequently generated with the re-scaled signal intensity distribution (panel D2).

Since OCT measures the intensity of back-scattered light from a sample, large objects like bacterial aggregates will scatter more strongly light than a single bacterium or EPS molecules and thus bacterial aggregates will yield a higher signal intensity than EPS molecules or water. This is demonstrated in Fig. 3, in which the OCT signal intensity taken of bacterial suspensions in an aqueous medium, is plotted as a function of the bacterial concentration in suspension, expressed as an optical density. Clearly, signal intensity increases linearly with increasing bacterial concentrations regardless of the strain or species involved. Therefore, the intensity distribution within a biofilm image can be expected to reflect bacterial presence, or the presence of (non-water-soluble) EPS molecules. In order to allow quantitative comparison of different biofilm images, the influence of the auto-scaling (see Fig. 1), needs to be eliminated to which end we developed a new, re-scaling method, as explained in Fig. 4. Note that in the re-scaling method proposed, the average signal intensity in an image of a biofilm-free substratum is used as the new maximal signal intensity and adjusted to a value of 255 a.u. The biofilm-free substratum is preferred to this end, as it is much easier to focus upon than on a biofilm-covered substratum, especially when covered by a thin biofilm. For the widely different materials used in this study, average substratum signal intensities in absence of biofilm prior to re-scaling were 84, 85 and 83 a.u. for SS, glass, and polystyrene, respectively. The rescaled intensity of pixel i (I_i_) is

$${I}_{i}=\frac{({I}_{0}-{I}_{w})\times 255}{{I}_{s}}$$

where I_0_ is the original intensity associated with a pixel before rescaling, I_w_ is the average intensity of pixels representing water above the biofilm excluding the potential pixels that are regarded by Otsu thresholding as floating bacteria or bacterial clusters, I_s_ is the intensity of the biofilm-free substratum.

### Comparison of auto- and re-scaling methods for analysis of biofilm structure: Signal intensity as a function of height in the biofilm

The auto- and re-scaling based analyses were applied on OCT images of the four biofilm cases (see Table 1, Fig. 1). In order to determine the validity of both methods, results will first be compared with the expected structural properties of each of the biofilm cases.

**Figure 5:**
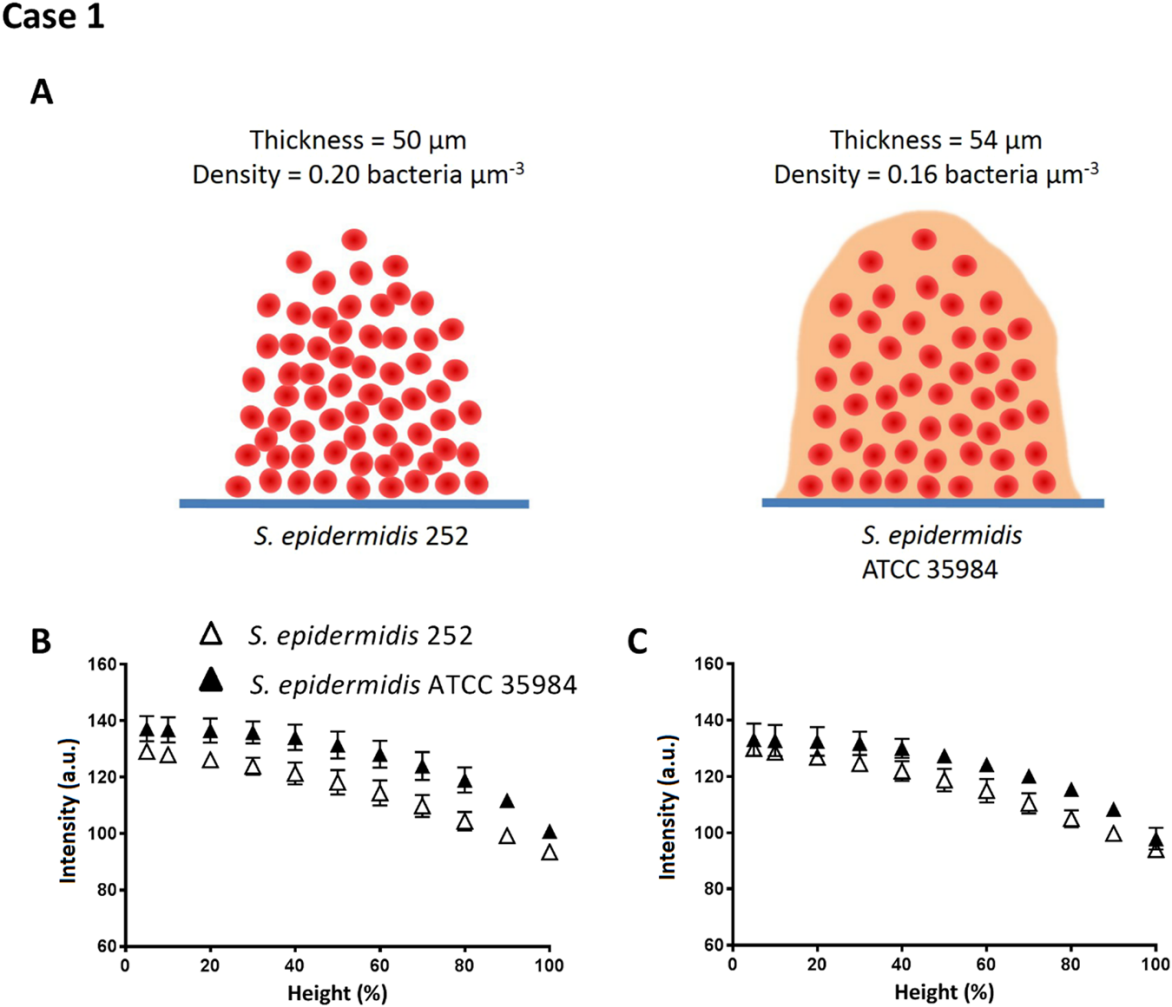
Signal intensity analysis of OCT images: biofilms of non-EPS producing *S. epidermidis* 252 and EPS producing *S. epidermidis* ATCC 35984. (**A**) Schematic presentation of non-EPS producing *S. epidermidis* 252 and EPS producing *S. epidermidis* ATCC 35984 biofilms (see Table 1) and their measured thickness (derived using Otsu thresholding of auto-scaled OCT images) and volumetric bacterial density, calculated from the biofilm thickness and enumeration of the number of bacteria in the biofilm. (**B**) Intensity as a function of biofilm height (%) for *S. epidermidis* 252 and *S. epidermidis* ATCC 35984 biofilms in auto-scaling analysis. (**C**) Same as (**B**), but now in re-scaling analysis. Error bars indicate standard deviations over triplicate experiments with separate bacterial cultures.

In order to evaluate the merits of the different methods with regards to EPS production in biofilms, *S. epidermidis* 252 (non-EPS producing) and *S. epidermidis* ATCC 35984 (EPS producing) were included (Fig. 1, case 1). Under the static growth conditions applied, both staphylococcal strains grew biofilms with a similar thickness of 50 µm (*S. epidermidis* 252) to 54 µm (*S. epidermidis* ATCC 35984). EPS producing *S. epidermidis* ATCC 35984 had slightly lower bacterial density (0.16 bacteria µm^−3^) than non-EPS producing *S. epidermidis* 252 (0.20 bacteria µm^−3^). These structural features are schematically summarized in Fig. 5A. Both auto- and re-scaling analyses (Fig. 5B,C, respectively) showed a significantly (one-tailed and paired student t-test with P < 0.05) higher average signal intensity in biofilm images across the height of EPS producing *S. epidermidis* ATCC 35984 than of non-EPS producing *S. epidermidis* 252, although significance was not observed when comparing intensities at a given height. Volumetric bacterial density of the EPS producing *S. epidermidis* ATCC 35984 was probably lowest due to volume occupation by EPS.

**Figure 6:**
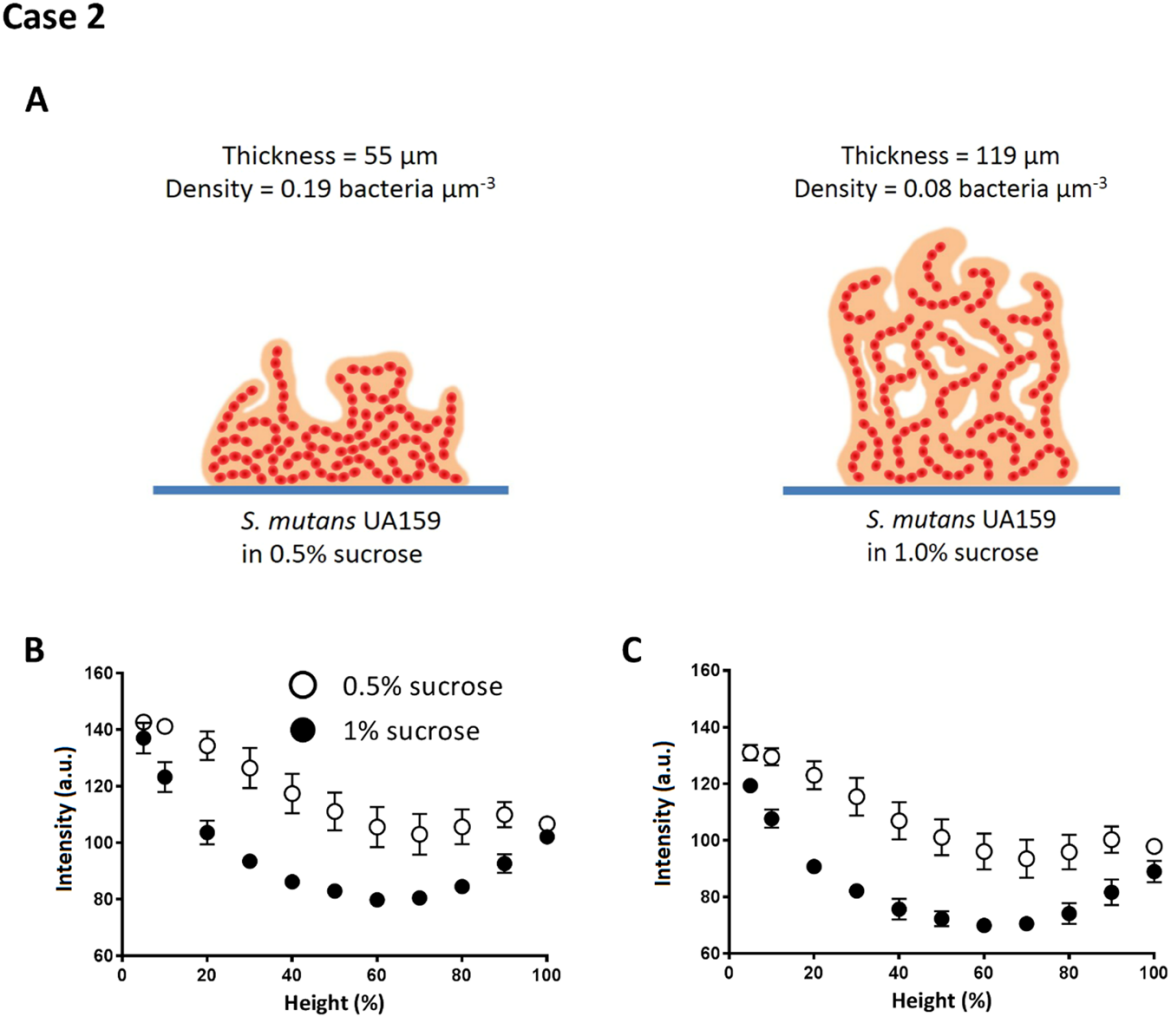
Signal intensity analysis of OCT images: *S. mutans* UA159 biofilms grown in medium with 0.5% or 1% sucrose added. (**A**) Schematic presentation of *S. mutans* biofilms grown in medium with 0.5% or 1% sucrose added (see Table 1) and measured thickness and volumetric bacterial density. (**B**) Intensity as a function of biofilm height (%) for *S. mutans* biofilms grown with 0.5% or 1% sucrose added in auto-scaling analysis. (**C**) Same as (**B**), but now in re-scaling analysis. Error bars indicate standard deviations over triplicate experiments with separate bacterial cultures.

Higher amounts of sucrose during growth of *S. mutans* biofilms yields biofilms with more water-filled channels (see Table 1)^31^. In our study, biofilms grown with 1% sucrose added were found to be twice as thick (119 µm), but with lower bacterial density (0.08 bacteria µm^−3^) than biofilm grown with 0.5% sucrose added. The above characteristics are shown schematically in Fig. 6A. Accordingly, OCT images of streptococcal biofilms grown with a higher amount of sucrose appeared on average significantly (one-tailed and paired student t-test with P < 0.05) with a lower signal intensity across the entire depth of the biofilms both in auto- and re-scaling analyses (Fig. 6B,C, respectively) due to the possession of more water-filled voids and pores. In addition, intensity analyses indicated that especially at heights in the middle of the biofilms, significantly more water-filled voids and pores are present.

**Figure 7:**
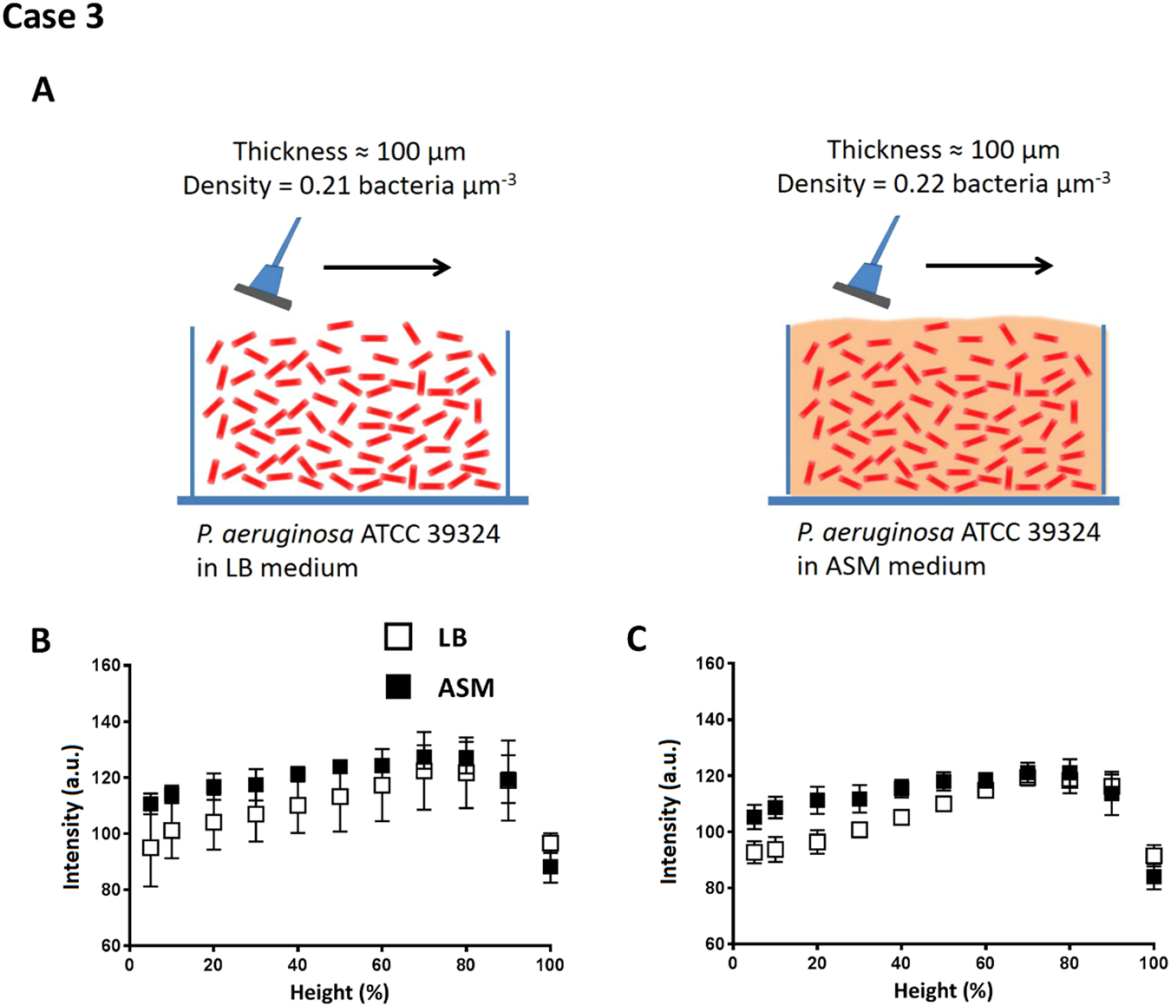
Signal intensity analysis of OCT images of *P. aeruginosa* ATCC 39324 biofilms grown in LB and ASM medium. (**A**) Schematic presentation of *P. aeruginosa* biofilms grown in LB and ASM medium (see Table 1) and measured thickness and volumetric bacterial density. Note that due to growth in a CDFF, thicknesses are identical. (**B**) Intensity as a function of biofilm height (%) for *P. aeruginosa* biofilms grown in LB or ASM medium in auto-scaling analysis. (**C**) Same as (**B**), but now in re-scaling analysis. Error bars indicate standard deviations over triplicate experiments with separate bacterial cultures.

Biofilms of *P. aeruginosa* strains (case 3) were grown in a CDFF to yield a thickness of 100 µm in LB and ASM medium. Biofilms grown in LB had similar bacterial density (0.21 bacteria µm^−3^) as in ASM (0.22 bacteria µm^−3^). Growth in ASM medium was expected to yield a more EPS-rich matrix than growth in LB medium^28^, as schematically indicated in Fig. 7A. However, auto-scaling analysis did not show any significant difference between intensity distribution across images of LB and ASM grown biofilms (Fig. 7B). Re-scaling analysis on the other hand (Fig. 7C), showed pseudomonas regions in biofilm images with significantly higher signal intensities (one-tailed and paired student t-test with P < 0.05) near to the substratum surfaces when grown in ASM than when grown in LB medium. This presents a clear advantage over re-scaling analyses compared to auto-scaling analyses. Potentially, scraper action has dragged EPS out of the outer regions of the biofilms.

**Figure 8:**
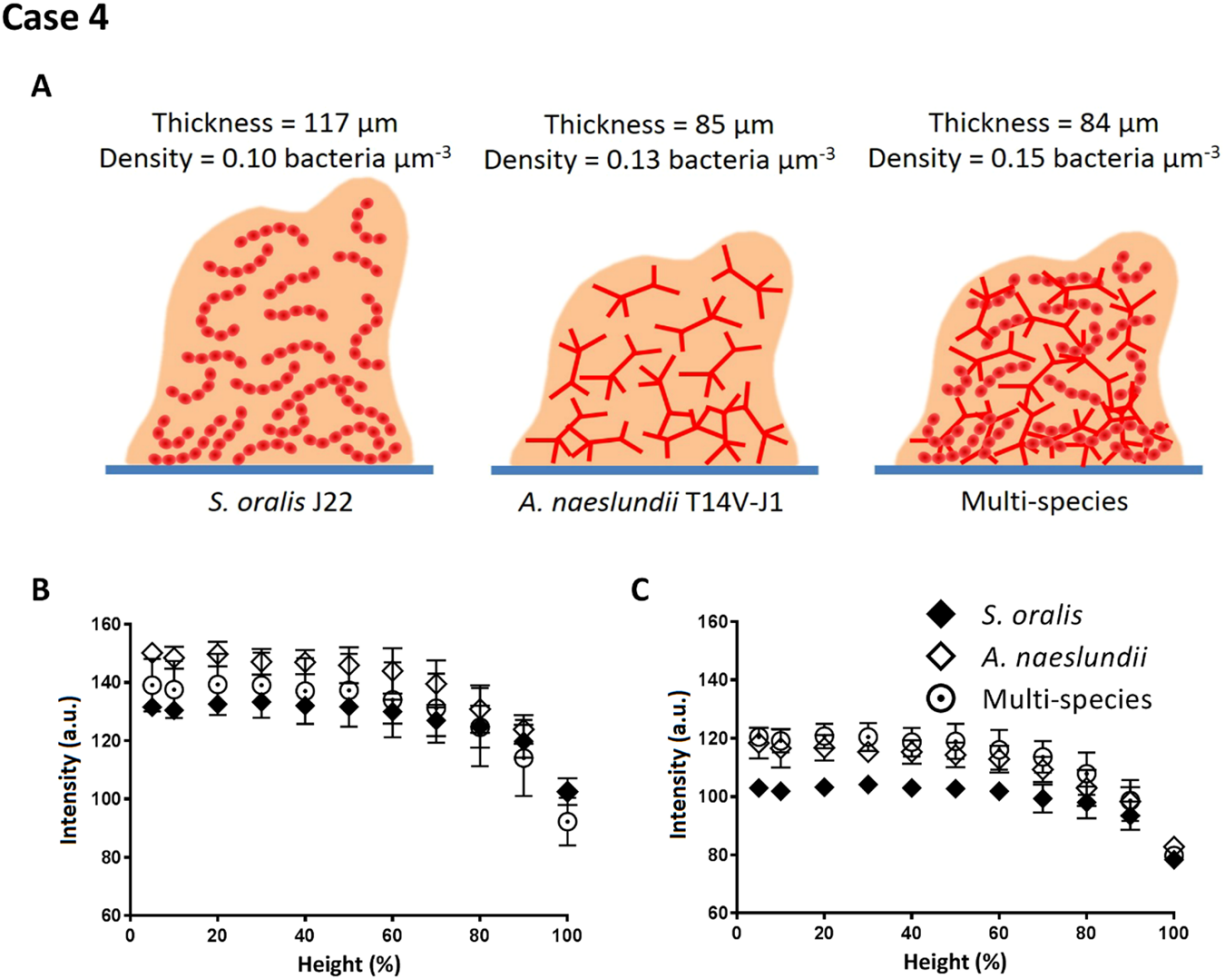
Signal intensity analysis of OCT images of single-species *S. oralis* J22 and *A. naeslundii* T14V-J1 biofilms and multi-species biofilms. (**A**) Schematic presentation of the biofilms for both single-species and the more compact multi-species biofilms (see Table 1) and measured thickness and volumetric bacterial density. (**B**) Intensity as a function of biofilm height (%) for both single-species and multi-species biofilms, in auto-scaling analysis. (**C**) Same as (**B**), but now in re-scaling analysis. Error bars indicate standard deviations over triplicate experiments with separate bacterial cultures.

Single-species and multi-species oral biofilms of co-aggregating pairs were grown, giving rise to variations in thickness, but most notably to a higher volumetric bacterial density in multi-species biofilms (see Fig. 8A for schematics) due to co-aggregation of *S. oralis* J22 with *A. naeslundii* T14V-J1 (see also Table 1), yielding less water voids or channels^32^. Image intensities across the depth of the biofilms obtained by auto-scaling analyses did not reveal any significant difference between single-species biofilms nor between single- and multi-species biofilms (Fig. 8B). On average across the depth of the biofilms, the re-scaling analyses showed the expected higher signal intensities in images of multi-species biofilms as compared with *S. oralis* J22 (P < 0.05, one-tailed and paired student t-test), but not with respect to *A. naeslundii* T14V-J1 probably because volumetric bacterial densities were similar for *A. naeslundii* and multi-species biofilms (Fig. 8C). Higher signal intensity in images of multi-species biofilms after re-scaling analyses was especially evident nearest to the substratum. This suggest a gradient in bacterial composition in multi-species biofilms across the depth of the biofilms, created because *A. naeslundii* was first seeded on the substratum surface after which the streptococci were allowed to co-adhere and grow in concert with the adhering actinomyces.

### Comparison of auto- and rescaling methods for analysis of biofilm structure: Relation between signal intensity and volumetric bacterial density in biofilms

**Figure 9:**
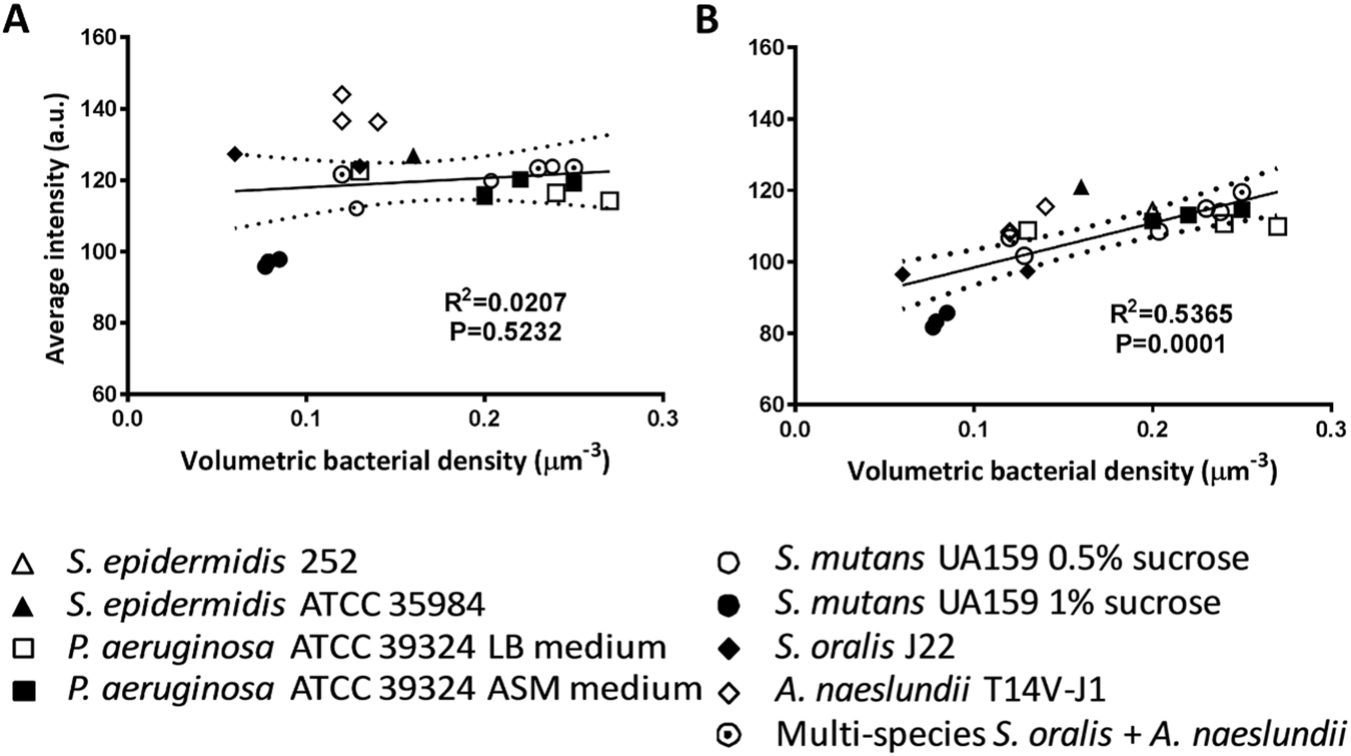
Average signal intensity in OCT images of biofilms as a function of the volumetric bacterial density for all individual biofilms grown of the different cases in auto- (panel A) and re-scaling analysis (panel B). Drawn lines represent the best fit to an assumed linear relation with correlation coefficient R^2^ and significance of the slope P indicated. Dotted lines represent 95% confidence intervals.

In order to make a comparison between auto- and re-scaling methods of OCT images, volumetric bacterial densities of all biofilms are presented as a function of the average signal intensity over each OCT image of the biofilms, as calculated from auto- (Fig. 9A) and re-scaling (Fig. 9B) analyses. Eliminating auto-scaling by our proposed re-scaling method, yielded a significant linear relation between signal intensities and volumetric bacterial density in a biofilm with signal intensity increasing with increasing density. Auto-scaling of OCT images clearly does not yield statistically reliable, quantitative conclusions to be drawn across different biofilms and different substratum surfaces regarding volumetric bacterial density. Thus, it can be concluded that the proposed re-scaling removed the impediment associated with auto-scaling to quantitatively compare individual images. Accordingly, the wide variety of different strains and species involved in the relation between signal intensity and volumetric bacterial density (Fig. 9B) enables to derive volumetric bacterial densities in a biofilms from the re-scaled OCT intensity in biofilm images, without need to culture.

Within the current collection of biofilms, volumetric bacterial densities of the different biofilms varied by a factor of 3, which covers the range of density variation for different biofilms in the literature^33–35^. Since distances between biofilm inhabitants have been reported to range between 1 and 3 µm^36^, volumetric bacterial densities in biofilms are generally low^37^, as compared e.g. with the closest hexagonal packing of a 1 µm diameter sphere yielding a volumetric density of 1.5 µm^−3^. This confirms that most volume in biofilms is occupied by water with or without dissolved EPS.

The relation between signal intensity and volumetric bacterial density presented in Fig. 9B is obtained using averaged signal intensities over the entire biofilm height. This was needed in order to relate signal intensity with numbers of bacteria, which could not be determined as a function of height in the biofilm. In most biofilms, volumetric bacterial density decreases towards the top of a biofilm, where a more open structure prevails. Structural differences across the depth of a biofilm are strain-dependent and furthermore arise from different nutrient availability, physical stresses and many other environmental factors. However, based on Fig. 9B, local volumetric bacterial densities across the depth of a biofilm can now be derived from signal intensity distributions. This may be a major asset in biofilm research, particularly since antimicrobial exposure of biofilm has been described to be accompanied by an increase in signal intensity in OCT images^21^ that can now be more solidly interpreted.

Although the current study indicates that light scattering by bacteria is the overriding cause of signal intensity distributions in OCT images, else a relation as in Fig. 9B would not develop, a disadvantage of OCT remains that also with the current methodology, the chemical composition of biofilm (matrix) cannot be directly determined.

## Conclusions

A re-scaling method is presented that undoes the effects of auto-scaling in OCT images and therewith allows to compare signal intensity distributions in different OCT images of different biofilms, including single- and multi-species ones (coccal and rod-shaped) and on different substratum materials. Qualitatively, both auto- and re-scaled signal intensities in biofilm images as a function of depth in different biofilms could be interpreted in line with biofilm characteristics expected on the basis of literature for the different biofilms. However, specific features of pseudomonas and oral multi-species biofilms were more prominently expressed after re-scaling. Average re-scaled signal intensities in OCT images of different biofilms increased linearly with independently determined volumetric bacterial densities in the biofilms, therewith quantitatively validating the re-scaling method proposed. Theoretically^16^, the signal-to-noise ratio of the back-scattered light declines along the optical axis of the OCT device, which might create an impact of biofilm thickness on the measured light intensities. However, since signal intensity in our OCT images remained constant over the depth of 300 µm homogeneous agar layers (Fig. 2), we believe that our proposed method will be valid in biofilms of at least this thickness. Herewith the proposed re-scaling of the signal intensity distribution in OCT images of biofilms significantly enhances the usefulness of OCT biofilm imaging, as applicable on an entire biofilm image, but as can also be applied on image sections, representing e.g. high density bacterial clusters.

## Data Availability

Data are available on request.
